# Metabolomics-Based Selection of Biostimulant and Biocontrol Microbial Consortia

**DOI:** 10.3390/metabo16080582

**Published:** 2026-08-17

**Authors:** Polina Volkova, John M. Wong, Jacqueline Wong

**Affiliations:** TLC Products, Inc., Westlake, OH 44145, USA; johnmichaelwong@gmail.com (J.M.W.); jacqueline@tlc-products.com (J.W.)

**Keywords:** plant growth-promoting bacteria, PGPB, multiple use principle, *Bacillus*, *Rhodopseudomonas palustris*, nitrifiers, AOB, NOB

## Abstract

Microbial biostimulants and microbial plant protection products overlap in biological function, creating both R&D opportunities and regulatory challenges. In particular, multi-strain bacterial consortia may simultaneously affect nutrient mobilisation and abiotic stress tolerance, induce resistance, and demonstrate direct antagonism against phytopathogens. This multifunctionality complicates early product development because strain identity alone is sometimes insufficient in predicting product function, efficacy, or the most appropriate regulatory and claims strategy. Here, we used non-targeted LC-MS metabolomics as a hypothesis-generating tool to support formulation decisions for microbial consortia. Three bacterial consortia were compared: a full soil-oriented consortium C1 containing *Bacillus* spp., *Rhodopseudomonas palustris*, *Nitrosomonas europaea*, and *Nitrobacter winogradskyi*; a *Bacillus*-only consortium C2 intended for foliar stress-resilience applications; and a *Bacillus*-only consortium C3 grown with a chitin-related inducer to promote biocontrol-associated metabolism. Metabolomic profiling revealed clear differences between formulations. The full consortium C1 showed higher relative abundances of features putatively associated with biofertilising and growth support, whereas the *Bacillus*-only consortium C2 contained features putatively associated with biocontrol and induced resistance that were not detected in C1 under the applied criteria. The addition of the chitin-related inducer (C3) did not yield a completely distinct metabolite profile but increased the relative abundance of selected features putatively associated with biocontrol, while decreasing features putatively annotated as auxin-related or associated with abiotic stress responses. These results suggest that non-targeted metabolomics can help differentiate metabolic profiles putatively associated with biostimulant- and plant-protection-oriented formulations and thereby support prioritisation before extensive greenhouse or field testing. By linking formulation, medium composition, and microbial interactions to measurable metabolic signatures, metabolomics provides an evidence-based, hypothesis-generating framework for formulation development and the prioritisation of subsequent efficacy trials.

## 1. Introduction

Microbial biostimulants have become a rapidly expanding part of sustainable agriculture, aiming to improve nutrient use efficiency, stress resilience, and crop performance while reducing dependence on conventional agrochemicals and helping agriculture respond to climate and environmental constraints [1,2]. Despite this momentum, major knowledge gaps still limit the robust development and deployment of microbial stimulants. Product performance is strongly context-dependent [3], varying with crop genotype, soil properties, climate, management, and the resident microbiome, while many formulations still suffer from inconsistent efficacy and incomplete mechanistic characterisation. This uncertainty complicates product optimisation, field performance prediction, dose selection, and the design of formulations that remain reliable across contrasting environments and agronomic scenarios.

These challenges are exacerbated when microbial products are based on bacterial mixtures rather than single strains. Microbial consortia are attractive because they can combine complementary functions, including nutrient mobilisation, hormone modulation, stress buffering, and pest or pathogen suppression [4]. This biological multifunctionality is directly relevant to regulations. Recent analyses of the European framework emphasise that strict functional separation between plant biostimulants and plant protection products is often biologically artificial, as microorganisms may simultaneously support plant nutrition, abiotic stress tolerance, and the direct or indirect reduction in damage caused by pathogens and pests [5]. The current EU multiple-use debate, therefore, reflects a deeper scientific issue: the same organism, or the same consortium, can contribute to several agronomically relevant functions depending on formulation, dose, context, and claimed use, even though regulation still assigns products to distinct legal routes on the basis of intended function and claims. In this sense, regulatory ambiguity is not merely a legal problem, but also a symptom of insufficient mechanistic resolution in product development.

Modern omics approaches offer a way to move beyond this empirical bottleneck. Multi-omics and advanced phenotyping are increasingly being used to shift microbial product development from broad screening toward mechanistically informed design, enabling researchers to connect product composition with host responses, environmental performance, and target agronomic outcomes [6,7]. Non-targeted metabolomics is a useful tool helping to dissect the chemical composition of complex microbial products, including exo-metabolites, intracellular metabolite repertoires, postbiotic fractions, and interaction-driven chemistry in consortia, thereby helping to optimise formulation [8,9]. The comparison of single strains with a commercial *Bacillus* consortium showed that the consortium had a distinct chemical profile, including relatively high surfactin abundance [10]. Extracellular metabolite profiling showed that cross-feeding between *Priestia megaterium* and *B. licheniformis* drives distinct metabolic reprogramming [11], and volatile organic compound (VOC)-mediated interactions between these microbes led to changes in both endo- and exo-metabolomes during co-culture [12]. This variation in product metabolic composition due to component variation gives important insights during the product R&D phase and helps to decide if the product should be registered and marketed as a plant protection product or as a biostimulant, as plant-associated microbes are evolutionarily capable of performing both functions, and, in principle, the same bacterial component can appear in different products under different legal frameworks. There are also recent strain-level examples showing that true dual functionality can exist. *B. velezensis* EM-A8 was reported to provide both disease suppression of *Exserohilum turcicum* and growth enhancement in maize [13]. Another *B. velezensis* strain, CNPMS-22, was presented as both a biocontrol agent against pathogenic fungi and a plant growth promoter [14]. Finally, an olive-tree endophyte, *B. velezensis* Amfr20, was likewise proposed as a future ecological biopesticide and/or biostimulant [15].

In this short communication, we provide an example of a hypothesis-generating proof-of-concept study using a non-targeted metabolomics approach for product development, in which we analyse different consortia and media compositions to define the most suitable product function for future greenhouse and field tests.

## 2. Materials and Methods

### 2.1. Consortia Preparation

In this work, we compared 3 consortia developed by TLC Products, Inc. (Westlake, OH, USA) and used across many geographical regions and crop species:

Consortium 1 (C1)—six bacterial species, *Bacillus subtilis* TLC, *B. velezensis* TLC, *B. licheniformis* TLC, *Rhodopseudomonas palustris* TLC, ammonia-oxidising bacteria *Nitrosomonas europaea* TLC, and nitrite-oxidising bacteria *Nitrobacter winogradskyi* Nb-255. According to various field reports and internal case studies, C1 has increased biofertilization capacity and stimulates growth as a soil-drench application.
Consortium 2 (C2)—contained only *B. subtilis* TLC, *B. velezensis* TLC, and *B. licheniformis* TLC. According to various field reports and internal case studies, C2 is suitable for foliar applications to promote stress resilience.
Consortium 3 (C3)—contained only *B. subtilis* TLC, *B. velezensis* TLC, and *B. licheniformis TLC*. Growth medium contained an inducer—a chitin-related polymeric compound. According to various field reports and internal case studies, C3 is suitable for foliar application to ensure plant protection.

All consortia contained 1 × 10^9^ CFU/g of each strain and were grown for 3 days in 500 mL Erlenmeyer flasks, in the liquid medium containing the following components: yeast extract (0.75 g/L), D-glucose (5 g/L), sodium acetate, anhydrous (6 g/L), MgSO_4_ × H_2_O (0.60 g/L), K_2_HPO_4_ (1.5 g/L), NH_4_Cl (0.6 g/L), and MnSO_4_ × H_2_O (0.05 g/L). Consortium 3 additionally contained a chitin-related polymeric compound (0.05%). Erlenmeyer flasks were continuously shaken on an orbital shaker (175 rpm) at 27 °C for 72 h. After three days of incubation, the inoculated medium was transferred to 15 mL Falcon tubes and centrifuged in an SCI-412 Clinical Lab Centrifuge (SCILOGEX, LLC, Rocky Hill, CT, USA) at 4500 rpm (~2500 g). The centrate was further analysed in the metabolomic pipeline. Four independent replicates were used for each consortium. The use of four biological replicates per formulation represents a limitation of this study, particularly for detecting subtle differences after multiple-testing correction; consequently, the identified metabolic patterns should be considered exploratory and require further validation.

### 2.2. Metabolomic Analysis

An amount of 1 mL of sample was lyophilised, 300 μL of 80% methanol (all reagents obtained through Sigma-Aldrich, St. Louis, MO, USA) was added, and the sample was then vortexed for 30 s followed by sonication in an ice-water bath for 30 min. The sample was kept at −20 °C for 1 h, vortexed for 30 s, and centrifuged for 10 min at 12,000 rpm, 4 °C. Finally, 5 μL of 0.14 mg/mL of DL-o-chlorophenylalanine was added to 200 μL of supernatant and then filtered through a 0.22 μm filter.

Separation was performed by a Vanquish Flex UPLC combined with a Q Exactive plus (Thermo Fisher Scientific, Germering, Germany) equipped with a heated ESI source. A Hypersil gold C18 column (Thermo Fisher Scientific, Runcorn, UK, 100 × 2.0 mm × 1.9 μm) was used for LC separation and the mobile phase was composed of solvent A (0.05% formic acid in water) and solvent B (acetonitrile) with a gradient elution (0–1 min, 5% B; 1–12.5 min, 5–95% B; 12.5–13.5 min, 95% B; 13.5–13.6 min, 95–5% B; 13.6–16 min, 5% B). The mobile-phase flow rate was 0.3 mL/min. The column temperature was maintained at 40 °C, and the sample manager temperature was set at 4 °C. Mass spectrometry parameters in ESI+ and ESI− mode were as follows: ESI+ (ESI−): heater temperature, 300 °C; sheath gas flow rate, 45 arb; aux. gas flow rate, 15 arb; sweep gas flow rate, 1 arb; spray voltage, 3.0 (3.2) KV; capillary temperature, 350 °C; S-lens RF level, 30 (60)%. Each biological and QC sample was injected at 2 µL and analysed in separate positive- and negative-ionisation runs using full-scan MS with dd-MS^2^ acquisition. Full-scan spectra were acquired over *m*/*z* 70–1050 at 70,000 FWHM resolution, with an AGC target of 3 × 10^6^ and a maximum injection time of 100 ms; data-dependent MS^2^ acquisition used a Top10 method, 17,500 FWHM resolution, an AGC target of 1 × 10^5^, a maximum injection time of 50 ms, a 1.7 *m*/*z* isolation window, and HCD fragmentation with stepped normalised collision energies of 15, 30, and 45.

Raw LC-MS data were processed in MS-DIAL (v5.5.250820, NET48) after installation of Thermo MSFileReader 3.0 SP2. A total of 12 positive-mode and 12 negative-mode Thermo .raw files were analysed, together with 4 QC files for each ionisation mode. Positive and negative datasets were processed separately; raw files were imported into a new MS-DIAL project, and biological replicates were grouped accordingly. Peak annotation was performed using the MoNA-export-LC-MS_Spectra.msp, MassBank_RIKEN.msp and LipidBlast libraries. For ESI+ data, a restricted adduct list was applied, retaining mainly [M+H]^+^ and [M+Na]^+^, with [M+K]^+^ included only when relevant. After alignment, the aligned feature table was exported and signal intensities were normalised in the Data Visualisation module using the internal standard DL-o-chlorophenylalanine (Target ID set to 1). The final list of metabolites, along with their respective retention times and *m*/*z* ratios, is presented in Table S1. Compounds with MS/MS spectral library matches were reported as annotated metabolites. Compounds assigned based on accurate mass and other MS1-level evidence, but lacking an MS/MS library match, were designated putative metabolites.

We acknowledge that normalised LC–MS feature intensities are semi-quantitative and do not represent absolute metabolite concentrations. Nevertheless, they provide an appropriate basis for our primary objective of comparing relative feature abundance across the different consortia analysed under identical experimental and analytical conditions. Throughout the manuscript, metabolite names refer to putative annotations. For readability, the term “putative” is not repeated before every metabolite name (only for compounds lacking an MS/MS library match), but this omission should not be interpreted as confirmation of metabolite identity.

### 2.3. Data Analysis

Further data analysis was performed in Metaboanalyst 6.0 [16]. Applied filters: RSDs > 20% compared to QCs filtered out; low-variance filter—IQR (40%); low-abundance filter—median intensity value. Data were normalised by median, log-transformed, and auto-scaled. Principal component analysis (PCA) was applied to the processed feature-intensity matrix as an unsupervised method to visualise overall variation among samples. For each pairwise comparison, fold change was calculated as the ratio of the mean feature intensities between groups. Volcano plots combined log_2_ fold change with −log_10_-transformed *p*-values obtained using an unpaired two-sample *t*-test; features were considered differentially abundant at FC ≥ 2. For comparisons between C1 and C2, *p*-values were adjusted using the Benjamini–Hochberg false discovery rate correction. For comparisons between C2 and C3, raw *p*-values are reported because the more subtle differences did not remain significant after FDR correction; these results were therefore interpreted as exploratory.

## 3. Results

**Figure 1:**
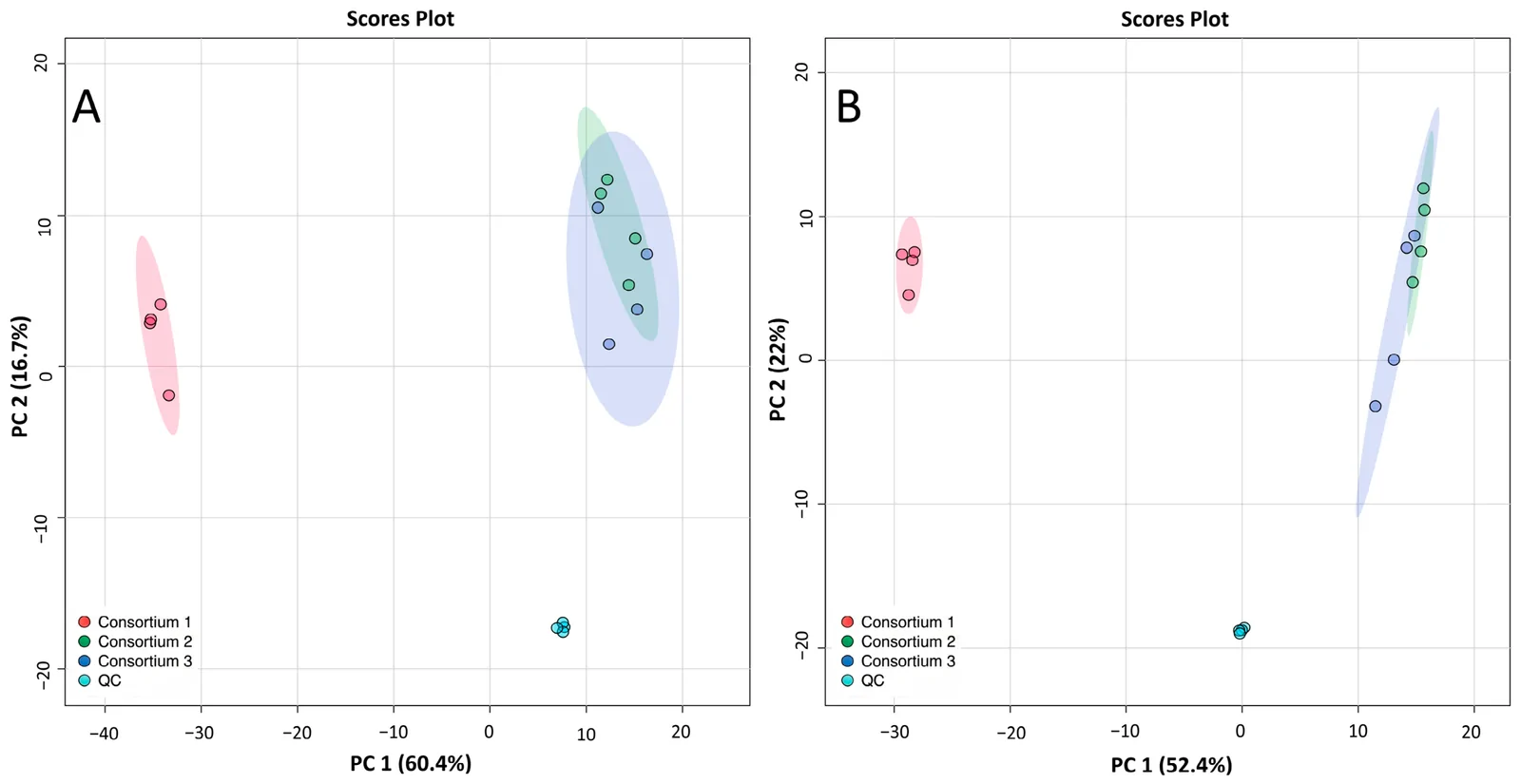
PCA score plots of metabolomic profiles of different consortia in positive (**A**) and negative (**B**) ionisation modes. PERMANOVA for negative mode: F-value: 444.12; R^2^: 0.99107; *p*-value (based on 999 permutations) = 0.001. PERMANOVA for positive mode: F-value: 371.65; R-squared: 0.98935; *p*-value (based on 999 permutations) = 0.001.

Optimisation of our consortia aimed at different functions. The C1 consortium, focused on quality traits and biofertilisation functions, included *Bacillus* spp. for growth promotion, *R. palustris* with strong biofertilisation capabilities, and a combination of *N. europaea* and *N. winogradskyi* to support nitrogen turnover and reduce nitrogen fertiliser requirements. The C2 consortium for foliar application, aimed at alleviating abiotic stress, contains a mix of *Bacillus* strains. Lastly, the C3 consortium explored the biocontrol capabilities of C2 *Bacillus* strains by varying formulation. PCA of non-targeted metabolomics results revealed that C1 was distinctly different from *Bacillus*-only consortia (Figure 1). At the same time, the addition of the chitin-like inducer in C3 led to the increased variation in compound concentrations compared with C2 without the inducer (Figure 1).

**Figure 2:**
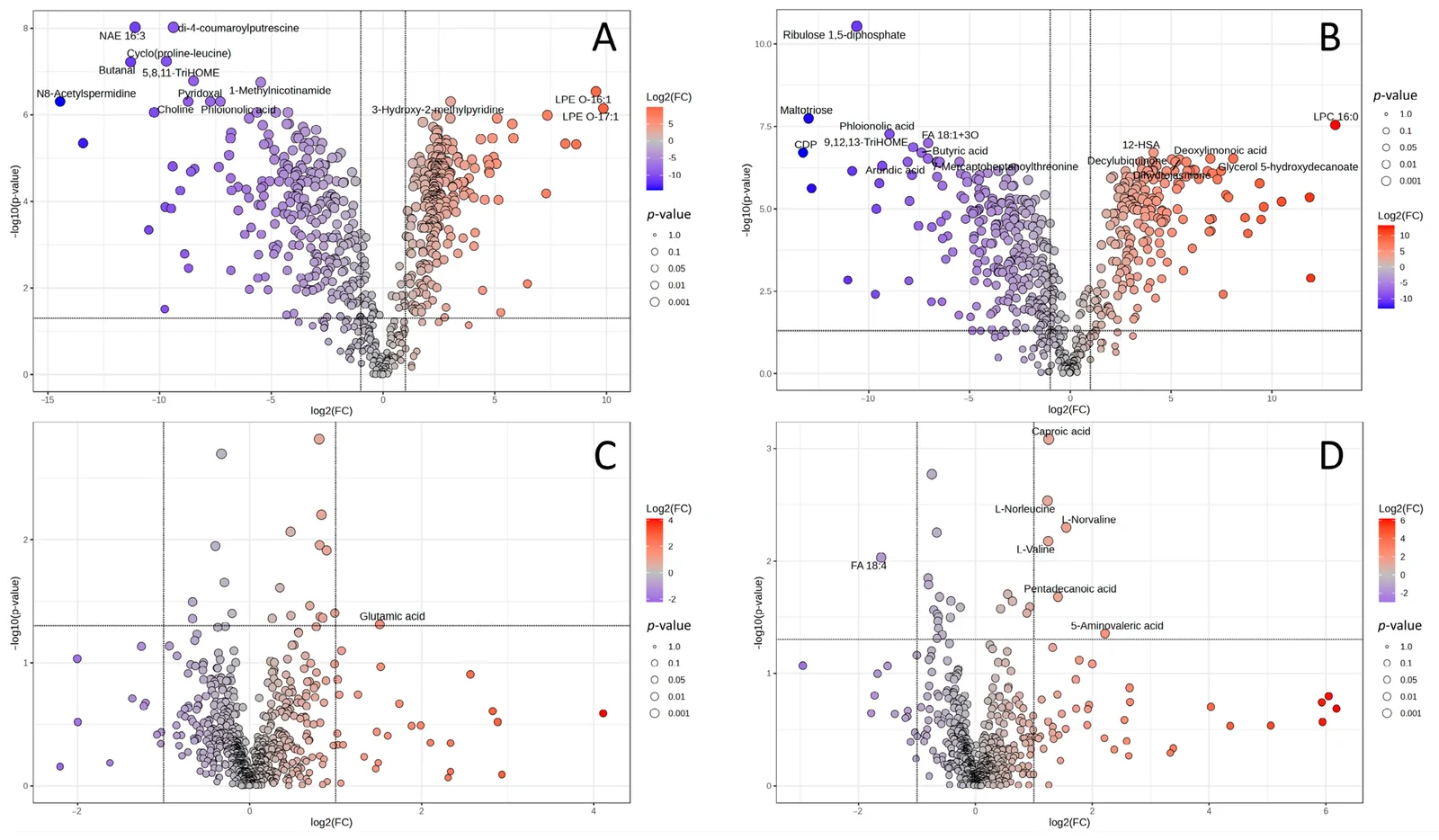
Volcano plots of significant differences in metabolite abundance. Differentially abundant metabolites are shown for the full consortium 1 compared with *Bacillus*-only consortium 2 in positive (**A**) and negative (**B**) ionisation modes; consortium 3 (with the inducer) compared with consortium 2 in positive (**C**) and negative (**D**) ionisation modes. The names of the top differentially abundant metabolites are shown (*p* < 0.05 after Benjamini–Hochberg FDR correction for (**A**,**B**); raw *p* < 0.05 for (**C**,**D**)). Log_2_FC direction: C1/C2 and C3/C2; positive values indicate higher abundance in C1 or C3, respectively. 12-HSA: 12-hydroxystearic acid; CDP—cytidine diphosphate; FA 18:1+3O—oleic acid; FA 18:4—stearidonic acid; LPC—lysophosphatidylcholine; LPE—lysophosphatidylethanolamine; NAE 16:3—N-hexadecatrienoylethanolamine.

To further explore the functional differences, we analysed combined univariate volcano plots of the fold changes of relative feature abundances between C1 and C2 (Figure 2A,B). Additionally, a list of differentially abundant metabolites is presented in Table S2.

Among the most increased differentially abundant metabolites in the full consortium C1 compared to C2 were 3-hydroxy-2-methylpyridine, lysophosphatidylethanolamine (LPE)-O-16:1, LPE-O-17:1, lysophosphatidylcholine (LPC) 16:0, 12-hydroxystearic acid (12-HSA), decylubiquinone, dihydrojasmone, deoxylimonoic acid, and glycerol-5-hydroxydecanoate. Metabolites that were the least abundant in the C1 compared to C2 include N-acylethanolamine (NAE) 16:3, di-4-coumaroylputrescine, cyclo(proline-leucine), butanal, N8-acetylspermidine, pyridoxal, 1-methylnicotinamide, choline, phloionolic acid, maltotriose, fatty acid (FA) 18:1, ribuloso-1,5-diphosphate, cytidine diphosphate (CDP), butyric acid, and 7-mercaptoheptanoylthreonine.

A set of features and their respective putative metabolites was not detected under the applied criteria in C1, but was detected in C2, and vice versa. Compared with the *Bacillus*-only C2 consortium, C1 contains fewer biocontrol-related and growth-inhibitory metabolites, but higher levels of LPE and LPC (Table 1).

## 4. Discussion

Several recent studies directly compared applications of multi-strain plant growth-promoting bacteria (PGPB) consortia or co-inoculation against single-strain applications. Consortia usually outperform single strains when the strains are functionally complementary and mutually compatible, and this effect has been shown across various crops, such as salt-stressed wheat [17] and rice [18], drought-stressed maize [19], or unstressed maize [20] and tomato [21]. However, some counterexamples can also be found in the literature. For instance, consortium products did not always improve yield beyond the best single *Bacillus* strains in tomato, and one consortium even performed worse in one season [22].

### 4.1. Functional Differences of Soil-Drench Consortium C1 and Foliar Consortium C2

Three formulations had different metabolomic profiles (Figure 1). The analysis of the functional differences between C1 and C2 (Figure 2A,B, Table 1) revealed that in the full consortium C1, several agriculturally potent metabolites were not found under given analytical conditions, while present in the *Bacillus*-only consortium C2. Among them are stress-alleviating metabolites, such as the dipeptide leucylphenylalanine, which was found to be the most responsive in a drought-recovery study in tomato (~14.8-fold higher), alongside improved recovery outcomes [23]. Current reviews summarise that dipeptides in plants are increasingly linked to stress resilience, antioxidant and redox biology, and regulatory interactions with proteins [24,25].

However, the most striking differences were observed for putative biocontrol metabolites, indicating that the C1 consortium may be much less potent as a potential biocontrol product. The following putative metabolites discussed in more detail were found only in the C2 consortium. A recent article reported that 5,6-dimethylbenzimidazole (Table 1, C2) is an antifungal factor produced by an endophytic *Flavobacterium* strain that suppresses *Rhizoctonia solani* disease in sugar beet seedlings [26]. Sphinganine (Table 1, C2) and related molecules can trigger defence-associated responses and control aphid infestation by altering endogenous sphingolipids [27]. Dihydroisocoumarin and related compounds (Table 1, C2) have been documented to have antifungal activity in vitro [28]. Di-4-coumaroylputrescine (Table 1, C2) belongs to the phenolamides that plants accumulate under biotic stress, which are widely described as phytoalexin-like defence metabolites with antimicrobial and anti-herbivore roles [29].

A further biocontrol signature in C2 is cyclo(proline-leucine), a cyclic dipeptide (Table 1, C2; Figure 2A). In *Arabidopsis*, bacterial cyclic dipeptides including cyclo(L-Pro-L-Leu) induced resistance against *Pseudomonas syringae* infection [30] and promoted growth and root branching [31]. In cucumber and pepper, seed defence biopriming with autoclaved metabolites from *Bacillus gaemokensis* PB69 induced resistance against *P. syringae* pv. *lachrymans* under in vitro and field conditions, and fractionation identified cyclo(L-Pro-L-Leu) as a key elicitor [32]. *Pseudomonas sesami* BC42 produced isomeric cyclo(Pro-Leu), and specific isomers reduced conidial germination and impaired development of *Colletotrichum orbiculare*, leading to reduced lesion severity on cucumber leaves [33]. Cyclo(L-Pro-L-Leu) purified from a *Pseudomonas putida* strain showed activity against the root-knot nematode *Meloidogyne incognita*, with reported effects on mortality and egg hatching [34]. *Achromobacter xylosoxidans* produced cyclo(L-Pro-L-Leu), which inhibited aflatoxin production by *Aspergillus parasiticus* and fungal growth [35].

Although lacking those biocontrol metabolites, the C1 consortium had higher levels of putative signalling molecules known to induce plant stress adaptation (Table 1, C1; Figure 1A). LPEs are signalling lysophospholipids implicated in plant growth, development, and stress responses [36]. They delay senescence by inhibiting phospholipase D and are used in postharvest treatments [37]. A cyclic nucleotide guanosine-3′,5′-cyclic monophosphate (cGMP, Table 1, C1) is also widely discussed as a signalling nucleotide in plants, involved in both development and stress responses [38]. In *Arabidopsis*, a membrane-permeable cGMP derivative has been used to modulate auxin-related responses and root development [39], and exogenous application was reported to improve performance under salt stress [40].

Finally, the C2 consortium metabolome shows multiple signs of more active bacterial growth and medium exhaustion at the same incubation time compared with the C1 consortium, suggesting that the full C1 consortium may hypothetically balance *Bacilli* growth. Such metabolites as 2′-deoxy-AMP, deoxycytidine-5′-diphosphate, cytidine-5′-monophosphate, NADH, GDP, ribulose 1,5-diphosphate, cytidine-5′-diphosphate, and 5-phosphorylribose 1-pyrophosphate (Table 1) reflect general metabolism and are notably increased in the C2 consortium.

### 4.2. The Addition of an Inducer Shifts the Metabolic Profile of the Bacillus-Only Consortium Further to Biocontrol

Evidence suggests that immune activation is costly and is often associated with a growth and development penalty, which is why balancing growth and defence has become a central breeding problem [41,42,43]. While any microbial biostimulant might have some extent of disease suppression, the primary function of such products is to improve nutrition and stress resilience. Stronger biocontrol or induced resistance product properties are often accompanied by some degree of growth inhibition, developmental delay, biomass reduction, or yield penalty, especially when defence is constitutively or strongly activated rather than merely primed. Therefore, microbial biostimulants are unlikely to be used as unlabelled plant protection products merely because they show some degree of disease suppression. Their primary agronomic value lies in stimulating plant development, nutrient acquisition, and stress resilience, whereas strong biocontrol or induced-resistance profiles may counteract this purpose by diverting resources away from growth. We therefore explored whether there is a straightforward way to shift the product from a biostimulant to a plant protection function during production. The C2 consortium, consisting of *Bacillus* species, whose genetic capability to induce immune responses in plants is well known, was tested in two versions: without (C2) and with (C3) an inducer. The inducer is a polymeric compound resembling fungal signals that primes the bacteria in the consortium to inhibit plant diseases.

To explore the functional differences, we analysed combined univariate volcano plots of fold change between C3 and C2 (Figure 2C,D; Table S2). The addition of the inducer did not result in detection of any new features under the applied criteria, but relative feature abundance of certain putative biocontrol compounds increased, presumably shifting the product to the biocontrol range. Among those significantly increased (raw *p* < 0.05) in all replicates were glutamic acid (Figure 2C), caproic acid, pentadecanoic acid, 5-aminovaleric acid, and the amino acids norleucine, norvaline, and valine (Figure 2D). Glutamic and caproic acids are particularly interesting for their role in plant defence. Exogenous glutamate acts as a plant signal, particularly in roots, where it influences root apical meristem activity and root branching architecture [44]; in rice under arsenic stress, exogenous glutamic acid also improved photosynthesis and growth while reducing oxidative damage [45]. L-glutamate also shows wide biocontrol capabilities: in postharvest tomato fruit, L-glutamate treatment reduced grey mould caused by *Botrytis cinerea* [46]; in rice, root treatment with glutamate induced systemic resistance to rice blast *Magnaporthe oryzae* [47]; in *Pseudomonas protegens*, glutamate specifically enhanced biocontrol efficacy against *Pythium* root rot in cucumber [48].

Caproic (hexanoic) acid reduced disease in tomato caused by both a necrotrophic fungus and a bacterial pathogen [49,50,51]. Treating citrus plants with hexanoic acid several days before infection reduced *Alternaria alternata* disease incidence and lesion development and activated defence pathways [52,53]. In melon, soil drench applications were tested for resistance against Melon necrotic spot virus, with reduced disease severity reported [54].

Additionally, a large set of putative biocontrol-related metabolites with at least a 2-fold increase was revealed in the C3 consortium compared with the C2 (Table S2). Below, we highlighted putative biocontrol-related metabolites with the highest differential abundance. 2-hydroxyisocaproic (leucic) acid (log_2_(FC) = 5.745) is repeatedly reported as part of a suite of antifungal and antimicrobial organic acids produced by bacteria in applied agricultural contexts [55,56]. An in vitro study reported succinic acid (log_2_(FC) = 2.414) among the active organic acids tested against *Alternaria alternata* [57]. There is also peer-reviewed evidence indicating succinic acid can affect growth, spore germination, and pathogenicity traits of a soil-borne *Fusarium oxysporum forma specialis* [58]. Succinic acid application to the rhizosphere shifted the rhizosphere bacterial community and recruited a beneficial *Sphingomonas* strain, which helped achieve 100% *Ralstonia solanacearum* biocontrol efficacy in a greenhouse experiment [59]. Another organic acid, DL-p-hydroxyphenyllactic acid (log_2_(FC) = 2.360), was implicated in antagonism against *Fusarium culmorum* in a barley malt substrate [56]. Lactic acid (log_2_(FC) = 2.288) inhibited mycelial growth and sporangium production of *Phytophthora nicotianae* in vitro, and reduced disease index *in planta*, increasing defence-associated markers [60]. A mechanistic study identified D-lactic acid as a defence-priming agent associated with induced resistance in *Arabidopsis* against *P. syringae* pv. tomato DC3000 [61].

Isovaleraldehyde (log_2_(FC) = 5.498), or 3-methylbutanal, is emitted by *Bacillus* spp. as part of their VOC profile. For example, in a biocontrol strain *B. subtilis* CF-3, the overall VOC mixture containing 3-methylbutanal inhibited the peach brown-rot pathogen *Monilinia fructicola* and induced defence and antioxidant enzyme responses in peach fruit [62]. Hexanal (log_2_(FC) = 4.029) can also act as an antifungal volatile: 2-hexenal inhibited *Botrytis cinerea* [63] and imazalil-resistant *Penicillium digitatum*, reducing green-mould outcomes on citrus [64]. Myriocin-12-en (log_2_(FC) = 11.354) is an antifungal antibiotic that has also been reported in bacterial metabolomes, such as *B. amyloliquefaciens* LZN01, and possesses strong antifungal activity against *Fusarium oxysporum* f. sp. *niveum* [21]. Myriocin was shown to control postharvest infection of wheat grains by *Fusarium graminearum* and reported strong antifungal effects in wheat grains [65].

Exogenous spermidine (log_2_(FC) = 2.968) application usually improves germination and abiotic stress tolerance [66,67], but peer-reviewed evidence indicates that it can also suppress disease development. For example, work in flax reports that spermidine and spermine inhibited *Fusarium oxysporum*, supporting the idea that polyamines can contribute to disease suppression outcomes in certain contexts [68]. Another well-known stress-alleviating metabolite increased in the C3 consortium is betaine (log_2_(FC) = 2.506, [69]), but a pearl millet study also reported glycine betaine-mediated induced resistance against downy mildew (*Sclerospora graminicola*), including host defence activation and disease suppression [70].

Besides induction of biocontrol metabolites, the C3 consortium has decreased abundance of features putatively annotated as growth-promoting hormone auxins (Table S2, indole-3-butyric acid, log_2_(FC) = −1.015; indole-3-acetic acid, log_2_(FC) = −1.172) and certain metabolites related to abiotic stress relief, which were more abundant in the C2 consortium. Among them is 3,4-dihydroxybenzoic acid (log_2_(FC) = −1.767), or protocatechuic acid, which increased rice shoot elongation during flooding stress (20–22% higher than controls) and improved [71]. Caffeic acid (log_2_(FC) = −2.928), applied exogenously to NaCl-stressed soybean, reduced oxidative damage markers in nodules and reversed salinity-associated reductions in nodule leghemoglobin and nitrogenase activity [72]. Cucumber seedlings pre-treated with caffeic acid showed reduced ROS and lipid peroxidation under chilling, higher antioxidant enzyme activities and related transcript levels, and increased proline and soluble sugars [73].

## 5. Conclusions

Overall, these results support non-targeted metabolomics as a practical hypothesis-generating and formulation-screening tool for microbial product R&D. In complex consortia, potential functional differences between formulations cannot be reliably inferred from strain identity alone, because the same strains may produce different chemical outputs depending on consortium composition, medium composition, and induction conditions. Here, metabolomic profiling distinguished a soil-drench-oriented consortium, with higher relative abundances of features putatively annotated as signalling-associated metabolites, from *Bacillus*-only formulations, which showed higher relative abundances of features putatively associated with stress alleviation and biocontrol, while the chitin-related inducer shifted the metabolic profile of the *Bacillus* consortium further towards features putatively associated with plant protection. This suggests that metabolomics can help prioritise formulations for subsequent greenhouse and field testing, generate hypotheses regarding potential application routes, and identify candidate functional traits for validation in efficacy trials.

Non-targeted metabolomics is a helpful hypothesis-generating tool for implementing the multiple use principle, because microbial products often sit at the biological interface between nutrition, abiotic stress tolerance, induced resistance, and direct antagonism, whereas most regulations separate plant biostimulants and plant protection products according to intended function and claims. For example, Regulation (EU) 2019/1009 defines plant biostimulants in terms of nutrient-use efficiency, abiotic-stress tolerance, quality traits, and nutrient availability, while Regulation (EC) No 1107/2009 covers products intended to protect plants against harmful organisms. Recent regulatory analysis likewise argues that the strict separation between microbial biostimulants and microbial plant-protection products may be biologically artificial, because the same microorganism or consortium can express different functions depending on formulation and use context. Non-targeted metabolomics can serve as an exploratory tool for identifying formulation-associated metabolic signatures and generating hypotheses for subsequent functional validation. By linking formulation choices to measurable metabolite signatures, metabolomics may support R&D optimisation and inform subsequent regulatory positioning.

## Figures and Tables

**Table 1:** Metabolites detected exclusively in consortium 1 or consortium 2.

Metabolite	ESI+/−	C1	C2	Probable Agricultural Role
5-Phosphorylribose 1-pyrophosphate	−	no	yes	Cell disruption/lysis marker
LPE 15:0-d5	−	no	yes	
MGMG 18:3	−	no	yes	
2′-Deoxy-AMP	−	no	yes	Cell disruption/lysis marker
Deoxycytidine-5′-diphosphate	−	no	yes	Cell disruption/lysis marker
Cytidine-5′-monophosphate	−	no	yes	Cell disruption/lysis marker
NADH	−	no	yes	Cell disruption/lysis marker
GDP	−	no	yes	Cell disruption/lysis marker
Ribulose 1,5-diphosphate	−	no	yes	Cell disruption/lysis marker
Cytidine-5′-diphosphate	−	no	yes	Cell disruption/lysis marker
Maltotriose	−	no	yes	
Leucylphenylalanine	+	no	yes	Dipeptide. Stress resilience.
1,3-Cyclohexanedione	+	no	yes	
Thymine	+	no	yes	Cell disruption/lysis marker
Fusaric acid	+	no	yes	
Dimethylbenzimidazole	+	no	yes	Biocontrol
Putative Harman	+	no	yes	Growth inhibitor, Biocontrol
Putative C17-Sphinganine	+	no	yes	Biocontrol
Eleutheroside E	+	no	yes	
Dihydroisocoumarin	+	no	yes	Biocontrol
Di-4-coumaroylputrescine	+	no	yes	Stress resilience/Biocontrol
Cyclo(proline-leucine)	+	no	yes	Biocontrol
Putative NAE 16:3	+	no	yes	Stress resilience
Putative BUTANAL	+	no	yes	Growth inhibitor
N8-Acetylspermidine	+	no	yes	Growth inhibitor
LPC O-18:1	+	yes	no	Stress resilience
Guanosine-3′,5′-cyclic monophosphate	+	yes	no	Stress resilience
Putative NAE 22:5	+	yes	no	
LPE O-16:1	+	yes	no	Biocontrol
LPE O-17:1	+	yes	no	Biocontrol
LPE 16:0	−	yes	no	Biocontrol

Note: LPE—lysophosphatidylethanolamine; MGMG—monogalactosylmonoacylglycerol; NAE—(N-acylethanolamine).

## Data Availability

The data are available at MassIVE MSV000102721.

## Supplementary Materials

The following supporting information can be downloaded at https://www.mdpi.com/article/10.3390/metabo16080582/s1, Table S1. Retention times, *m*/*z*, and feature abundances for each replicate; Table S2. FC of metabolite concentrations between consortia.

## Abbreviations

The following abbreviations are used in this manuscript:

VOCs	Volatile Organic Compounds
PGPB	Plant Growth-Promoting Bacteria
FA	Fatty Acid
LPC	Lysophosphatidylcholine
LPE	Lysophosphatidylethanolamine
NAE	N-Hexadecatrienoylethanolamine
FC	Fold Change
